# Adoption Processes of Innovations in Health Systems: The Example of Telemedicine in Germany

**DOI:** 10.3390/healthcare12020129

**Published:** 2024-01-06

**Authors:** Yvonne Rauner, Harald Stummer

**Affiliations:** 1 Institute for Management and Economics in Health Care, UMIT TIROL—Private University for Health Sciences and Health Technology, 6060 Hall, Austria; harald.stummer@umit-tirol.at; 2 Faculty for Business Administration, Seeburg Castle University, 5201 Seekirchen am Wallersee, Austria

**Keywords:** implementation, telemedicine, adoption theory, eHealth, innovation

## Abstract

(1) Background: Individual adoption experiences represent important factors in implementing innovations. In the context of health systems, where the implementation of innovations aims to improve the quality of care, they provide an important basis for developing and adapting implementation strategies. (2) Methods: This study examines the adoption experiences of (tele-)medical experts (*n* = 13) using the example of telemedicine in the German healthcare system by means of a qualitative, guideline-based interview study. The interview guide, as well as the deductive–inductive analysis, is based on Rogers’ adoption theory. The transcription and analysis process was carried out according to Kuckartz. (3) Results: A total of 304 interview statements could be coded and assigned to the five main categories of persuasion, knowledge, implementation, decision and confirmation. More than half of all statements were coded under the main category persuasion, with its subcategories of convictions regarding the implementation of telemedicine (*n* = 89), international comparison of Germany’s development (*n* = 50), telemedicine as a way of optimizing resources (*n* = 22) and conviction to understand telemedicine as an overall system (*n* = 10). (4) Conclusions: This study provides insight into how the implementation of telemedicine in the German healthcare system is perceived by experts and allows for adjustments to the ongoing implementation strategy.

## 1. Introduction

Despite a plethora of existing implementation theories and approaches [1], there is still a lack of understanding of how to implement innovations in the context of health systems in a manner that promotes or improves the quality of care in a rapid, holistic and sustainable manner [2]. Several studies show that this can have serious implications for the quality of care and well-being of patients in healthcare systems. For example, it is known that the nonimplementation of medical guidelines can mean that not all patients receive the same quality of care, and this can have a negative impact on their health outcomes [3,4]. In the context of innovative treatment methods, such as telemedicine, it is also known that poor implementation of this treatment method can have a negative impact on patient outcomes [5]. This is also described in an OECD Health Working Paper from 2020: “Telemedicine can be both safe and effective, in some cases with better outcomes than traditional face-to-face care” [6]. Given these far-reaching effects of delayed innovation and implementation of telemedicine, it is of particular interest to study this phenomenon in health systems that are lagging behind.

The Digital Health Index can be used as a possible indicator of innovation implementation in health systems. It is composed of the dimensions of policy activity, technical implementation and readiness for networking and data use and actual data use and exchange, and thus, it allows an international comparison of the implementation of innovations in health systems [7]. As part of a special evaluation, 17 OECD countries were analyzed with regard to telemedicine in standard care. Telemedicine is an umbrella term for various types of medical care that share the fundamental concept of providing medical services to the public in the areas of diagnosis, therapy and rehabilitation and advice on medical decisions via remote (or delayed) locations [8]. The terms telehealth, telecare, eHealth, mHealth, digital health, connected health, digital care and remote care are used synonymously; the terms teleconsultation, telediagnostics, teletherapy and telemonitoring are subcategories. The Digital Health Index combines the different aspects of telemedicine, such as electronic health records, e-prescriptions or video consultations for health purposes, into a country-specific score [7]. While Estonia (score 81.9), Canada (score 74.7) and Denmark (score 72.5) are at the top of the Digital Health Index, Austria (score 59.81), Switzerland (score 40.6) and Germany (score 30) are at the bottom [7]. This means that Germany is a laggard in this ranking in terms of telemedicine implementation.

There are a number of reasons why health systems may lag behind in implementing innovation: different social protection systems (Beveridgean, Bismarkian and private economic systems), different perspectives on implementation (government, finance companies, manufacturers, etc.) and different priorities (public welfare, productivity, technology acceptance) can influence the implementation of innovation. For this reason, when implementing innovations in the healthcare context, it is not sufficient to communicate the positive consequences of an innovation [9,10]; it is necessary to systematically analyze the implementation environment using appropriate methods [10].

Another challenge in the health sector is that the nature of innovation is often not clearly defined. For example, healthcare innovations such as telemedicine often represent a hybrid of product, process and service innovations [11]. A telemedicine consultation does not only involve new software or hardware products but also changes the existing treatment and care processes and enables new forms of services, including new business and billing models [11]. This implies a variety of factors influencing product, process and service innovations.

Given the diverse characteristics of healthcare innovations, various theories and models can be used for the development of implementation strategies. From a scientific perspective, they can be divided into acceptance and adoption theories and models [12]. While acceptance-oriented theories and models (e.g., Theory of Reasoned Action (TRA) [13], Technology Acceptance Model (TAM) [14], Unified Theory of Technology Acceptance and Use (UTAUT) [15]) focus on individual, mental and behavioral determinants in relation to innovative products or technologies [16,17], adoption-oriented theories deal with the individual perceptions and attitudes of individuals.

One of the most frequently used adoption-oriented theories [18] is the adoption theory of Rogers [19]. It iteratively assigns the determinants of individual adoption decisions to a five-stage adoption process [20] consisting of “(1) knowledge, (2) persuasion, (3) decision, (4) implementation, and (5) confirmation” [19]. Based on this process flow, individual adoption processes in healthcare systems can be studied by analyzing existing knowledge, persuasion and developed decisions regarding acceptance or rejection of an innovation and implementation. These individual adoption decisions, in the context of a combined analysis of the adoption decisions of individual members, can provide information about the further diffusion of an innovation at the societal level [19,21]. Previous studies based on adoption theory suggest that the perceived relative advantage, observability and compatibility of an innovation [22], as well as previous behaviors, personal beliefs and values, may be possible predictors of technology adoption [22,23]. Further research on adoption topics such as user perceptions, perspectives and experiences, satisfaction, acceptance and adherence, usability and personal preparedness and awareness are available and underline the relevance of the perception, experience and readiness perspectives in the implementation of technological innovations in healthcare [24,25,26].

Despite evidence that using implementation theories can make a valuable contribution to the theoretical planning and design of implementation strategies in health, they have rarely been used for this purpose [27,28,29]. Their application to date has often been limited to the analysis of barriers to implementation [18] or the retrospective evaluation of implementation processes [27]. Rogers’ adoption theory [19] provides a scientifically accepted theoretical frame of reference from which individual adoption experiences can be explored, thus enabling analysis of the status quo of innovation in complex healthcare systems. This approach can be used to identify organizational and cultural characteristics of individual members of healthcare systems within thematic areas, which can serve as a basis for developing quantifying research methods. The starting point for the analysis of the adoption processes of innovations in healthcare systems is therefore not only the acceptance factors for innovations but also how and in what way the individual members of the healthcare system learn about innovations, what experiences they have had with them and what attitudes they have developed as a result.

Given the far-reaching implications for quality of care and patient wellbeing, implementing innovations in healthcare systems is of particular importance. The aim of this article is to contribute to the body of knowledge on the perception and attitude formation towards innovations in the context of healthcare systems. Due to the late adoption of telemedicine in the German healthcare system, physicians and telemedicine experts are interviewed about their individual adoption experiences with the aim of identifying context-sensitive issues as possible influencing factors on implementation strategies.

## 2. Materials and Methods

This study examines the adoption experiences of medical and telemedical experts with regard to innovations in the healthcare system using telemedicine as an example. Therefore, a four-stage interview guide based on Rogers’ theory of adoption and diffusion [19] was developed to elicit the individual adoption process, including experiences and perceptions (Supplementary Material, Guideline interview). The interview guide elicits individual understandings of the term telemedicine innovation, adoption characteristics such as “knowledge”, “persuasion”, “decision”, “implementation” and “confirmation” and diffusion characteristics. The developed interview guide was presented to an expert audience at a symposium and tested in advance to ensure argumentative validation. No modifications were necessary.

### 2.1. Sample

The participants were recruited from the professional network and via the snowball method according to selective sampling. Prerequisites for participation were a professional connection to medicine or telemedicine, including a professional activity in the medical or telemedical field, a minimum age of 18 years and an understanding of written and spoken German or English. The aim of the sampling was to select experts who were as homogeneous as possible in terms of the inclusion criteria but as heterogeneous as possible in terms of other characteristics such as gender, employment status (employed vs. self-employed), organizational form (inpatient vs. outpatient care) and expert background (medical vs. nonmedical) in order to obtain as much contrast in the statements as possible [30].

A total of 30 experts were contacted, of whom 15 agreed to participate in an interview. Thirteen interviews were conducted after written and verbal consent to participate was obtained, including consent for publication of anonymized data. The interviews were conducted via videoconference (*n* = 11) or telephone (*n* = 1). One interview took place simultaneously with 2 experts who knew each other and worked for the same employer so that a total of 12 transcripts with 13 participants could be analyzed.

In total, 13 professionals with various backgrounds in telemedicine and medicine were interviewed (M = 9; F = 4). There were 9 participants with specific telemedicine backgrounds from the following areas: telemedicine and telecommunications (*n* = 2), hospital and home automation management (*n* = 2), economic evaluation of telemedicine applications (*n* = 2), medical informatics (*n* = 1) and chief medical officer (*n* = 2). Four of these nine professionals were also licensed to practice medicine. In addition, there were 4 medical professionals without specific training in telemedicine from the fields of dermatology (*n* = 2), pediatrics (*n* = 1) and internal medicine (*n* = 1). The mean age of the respondents was 42.8 years (26 years ≤ x ≤ 61 years).

### 2.2. Data Collection

The data collection took place in August and September 2022, during the COVID-19 pandemic, with a heightened awareness of the issue of telemedicine. To ensure the objectiveness of implementation, all participants received a standardized, abbreviated explanation based on the information sheet they had already received in advance. Subsequently, a short verbal survey was carried out, and the current activity with a focus on tasks, previous points of contact with telemedicine and basic attitudes toward telemedicine (positive, negative or neutral) was collected and summarized in bullet points. This section lasted between 8–12 min. Afterward, an audio recording was started and the interview began. The average interview length was 18 min. The interviews were transcribed according to the simplified transcription rules of Kuckartz [31]. MaxQDA [32] was used to manage and analyze the data collected. The data collection was closed according to the principle of theoretical saturation after no new information could be collected [33,34].

### 2.3. Data Analysis

The transcripts were analyzed using the Kuckartz method of content-structuring qualitative content analysis against the background of the respective understanding of the term telemedicine [35]. The qualitative evaluation in the context of category formation was carried out deductively–inductively [35] against the background of adoption theory using the main categories “knowledge”, “conviction”, “decision”, “implementation” and “confirmation”, which were each inductively expanded by subcategories in the further analysis. The definitions of the main categories were given by Rogers [19]. The subcategories were determined inductively. For this purpose, the coded text passages of each main category were summarized in a table and arranged thematically. Further abstraction resulted in thematic fields that represent the subcategories. Thus, the entire text material was deductively coded according to the main categories and inductively expanded as part of an in-depth cross-document analysis. To ensure objectivity of interpretation, the coding was again checked for selectivity and plausibility by two independent, nonparticipating researchers after the analysis runs. At the end of the process, we carried out an overall analysis of the frequency of codes in all documents.

The Research Commission for Scientific and Ethical Issues of UMIT Tyrol, Austria, approved this study. This study was conducted in accordance with the tenets of the Declaration of Helsinki.

## 3. Results

In the following, the results of the short survey, the interviewees’ definition of telemedicine and the categories are presented.

### 3.1. Short Survey and Definition of Telemedicine

The short survey revealed that nine participants reported an overall positive attitude to telehealth innovations, two were neutral but concerned and one reported a change from initially negative to positive. Considering the definition of telemedicine, it turned out that the interviewees defined the term in a very similar way at the beginning of the interview with hardly any differences. The following statement gives an impression of the participants’ underlying understanding of the term telemedicine:

“*Telemedicine can actually be understood as any form of medicine at a distance. And that goes from apps, so I can help myself, dial in somewhere, somehow get some information, or talk to a doctor, or a nurse, to what we do: 24/7 video consultation of intensive care, so quite different, quite a wide range, but I would say everything that is medicine over some kind of distance, theoretically also on the phone. On the phone is also a form of maybe telemedicine. Yes, and I would perhaps try to open it up at least in that way. But I think it’s important to try to subdivide it further on*”.

(Int. 4, sec. 2)

Despite the common and clear understanding of the term telemedicine at the beginning of the interview, uncertainties arose in the course of the conversation regarding the clarity of the term. The following quotes illustrate this phenomenon:

“*To be honest, the term telemedicine is only marginally relevant to me. One imagines something, but it is a bit difficult to say what all belongs to it?*”

(Int. 1, sec. 27)

“*But whether that goes under telemedicine or just tele administration, I don’t know. I don’t know*”.

(Int. 1, sec. 27)

### 3.2. Categories

A total of 304 interview statements could be coded and assigned to the five main categories of persuasion, knowledge, implementation, decision and confirmation. Aggregated to the respective main category, the categories persuasion (*n* = 171) and knowledge (*n* = 96) were the most frequently coded categories. This was followed by implementation (*n* = 19), decision (*n* = 10) and confirmation (*n* = 8), respectively. The coding of the main categories of persuasion and knowledge was extended by subcategories as part of the inductive analysis. The category persuasion covers four subgroups: convictions regarding the implementation of telemedicine (*n* = 89), international comparison of Germany’s development (*n* = 50), telemedicine as a way of optimizing resources (*n* = 22) and conviction to understand telemedicine as an overall system (*n* = 10). The category knowledge is based on individual experience and explicitly acquired knowledge and includes the following subcategories: aspects about the existing telemedical innovations (*n* = 65), aspects of telemedicine in the context of structures in Germany (*n* = 24) and knowledge from abroad (*n* = 7).

#### 3.2.1. Persuasion: Convictions Regarding the Implementation of Telemedicine

The most frequently coded category, persuasion (*n* = 171), describes the convictions developed on the basis of previous and still ongoing experiences with regard to the innovation of telemedicine. Therefore, the subcategory convictions regarding the implementation of telemedicine describes common beliefs regarding telemedicine implementation. It included aspects of the implementation process, the status of the respective technologies and changes in patient care associated with telemedicine represented by the following statements:

“*Yes, well, you can see that at least now with the telematics infrastructure that I believe that without a certain political will, not much will happen*”.

(Int. 10, sec. 24)

“*And that is also what I am currently observing in our company, unfortunately. We have a good technology. We always get good feedback, and unfortunately we often hear from the doctors that I would like to use it, BUT we don’t have it, or we don’t have reimbursement, or we don’t have people sitting in front of the monitors 24/7 or or or and (...). The technologies are there, but everything around it is currently in short supply*”.

(Int. 2, sec. 33)

“*It must not lead to the fact that so to speak the physician is rationalized away. These fears also exist with some, no, but that he is simply more appreciated for his relevant tasks and then also to convey to the patients, so you are not just such an electronic guinea pig or otherwise somehow, but no, you are already fully integrated into a system, but valuable time is just concentrated on that, yes, your supervising doctor also has more time for you at the moment when it is really necessary*”.

(Int. 12, sec. 27)

“*And in the end, of course, it is about showing that telemedicine is not necessarily always a new procedure. One way or the other, that is just, those are known medical concepts, which are simply processed, with the help of technology and to make that of course also with the decision makers then understandable is so thus also our task, at the end accordingly*”.

(Int. 11, sec. 9)

#### 3.2.2. Persuasion: International Comparison of Germany’s Development 

This subcategory comprises statements concerning common beliefs regarding an international comparison of telemedicine development in Germany. It includes statements that highlight Germany’s unique characteristics in implementing telemedicine. They relate to attitudes regarding the development and implementation period as well as data protection. They are represented by the following statements:

“*And I don’t think we have to reinvent the wheel or put ourselves on some kind of pedestal that causes problems for us, when it works great in all the countries around us and also in Canada. So that is, I think, the biggest problem. Because [...] if that is not the case, then there are no innovation costs or willingness to invest in it, especially with this bad experience in the background. We just have to say we need that*”.

(Int. 12, sec. 16)

“*Yes, they’ve been developing for quite a long time, and so far it’s not working, and I remain skeptical about that*”.

(Int. 4, sec. 29)

“*So I think that where we started to build up this infrastructure was the right thing to do. Whether it was done so efficiently to spend so long on master data management and so on, so that the systems that are established today are actually already outdated again. And that we could have managed with digital identities and without, yes, without cards and connectors a long time ago. So there are certainly things that could have been done better*”.

(Int. 10, sec. 47)

“*And that is actually staggering for Germany, it has to be said. So, we have a lot of catching up to do*”.

(Int. 4, sec. 25)

“*You don’t always have to have such a prevention mentality as a data protection officer. As a rule, that’s what data protectionists have. They don’t want to do that, and they can’t really justify it*”.

(Int. 4, sec. 16)

#### 3.2.3. Persuasion: Telemedicine as a Way of Optimizing Resources and Conviction to Understand Telemedicine as an Overall System

These subcategories relate to the understanding dimension of telemedicine as a way of optimizing resources (3) and the understanding of an all-encompassing system (4). They are illustrated by the following statements:

“*So once the subject of location-independent care. I live in the country, I’m on vacation, I can still take advantage of the care or simply save the time it takes to get there, that’s the advantage*”.

(Int. 10, sec. 20)

“*We always differentiate according to electronic patient files or so, is a subarea, a module, which one would have, could need then in interaction with a video conference, in interaction with telemonitoring*”.

(Int. 11, sec. 17)

“*Otherwise, you always have local, great stand-alone solutions somewhere that may only work in subareas*”.

(Int. 11, sec. 29)

#### 3.2.4. Knowledge: Aspects of the Existing Telemedical Innovations

The knowledge category describes an individual’s knowledge of the existence and functioning of telemedicine innovation. Aspects of existing telemedical innovations mentioned included telesurgery (Interview 5, Section 19; Interview 2, Section 5), digital health applications (Interview 8, Section 9), telemetry (Interview 2, Section 11), robots (Interview 2, Section 13), telestroke units (Interview 4, Section 6) or platform ideas (Interview 2, Section 11).

#### 3.2.5. Knowledge: Aspects of Telemedicine in the Context of Structures in Germany

The topic aspects of telemedicine in the context of structures describes specific knowledge about the structures and framework conditions of telemedicine in Germany. It includes statements about the general attitude regarding telemedicine (Interview 13, Section 11), as well as specific problem situations, such as practical implementation (Interview 11, Section 42) or financing (Interview 6, Section 28). This subcode is illustrated by the following quote:

“*We have to do with all kinds of groups or representatives. So, whether these are patients or individual patients sometimes, for example, from the field of self-help, about also for the individual family doctor. But our main clientele are actually the various (...) players, i.e., associations, companies, politics. And you would actually have to acknowledge all of them individually*”.

(Int. 13, sec. 11)

#### 3.2.6. Knowledge: Knowledge from Abroad

The subcategory knowledge from abroad includes statements on the status and implementation of telemedicine in Scandinavia, Canada or the USA. For example, the digitization of healthcare per se in the U.S. and the implementation of telematics in Scandinavia are described (Int. 10, sec. 12). Another respondent reported from his own experience:

“*So, I can give you a simple example, I lived in Canada for a little less than three years now, from 2007 to 2010, in Vancouver. And I was fully integrated into the medical system. And what-, as a doctor I can also pick up my medication directly from the pharmacy. And they had my entire medical history. So they knew which medications I had taken and pointed out to me that I had an (…) intolerance. So that, that’s amazingly good*”.

(Int. 12, sec. 16)

#### 3.2.7. Implementation

The implementation category (*n* = 19) describes the period after which an individual has decided to adopt telemedicine innovation. It first describes the general implementation steps and experiences of the respondents, including specific implementation steps in the professional as well as individual context. In terms of general implementation steps, one respondent described that they deal with all areas of telemedicine but that telemonitoring currently predominates in terms of content (Int. 13, sec. 9). Another participant described the establishment of an interdisciplinary digital consultation platform for the exchange of information on rare muscle diseases in order to digitally process problem cases and to relieve the daily hospital routine (Int. 8, sec. 34). Specific implementation steps in the professional context were described in the areas of telemonitoring and teleconferencing (Int. 9, sec. 4; Int. 9, sec. 6; Int. 8, sec. 5; Int. 4, sec. 4) and telerehabilitation follow-up (Int. 12, sec. 5):

“*Tele-Intensive Medicine is what we do now. That means we’re advising and assisting intensivists 24/7 by bringing the data from the bedside to us in real time. Our command center with artificial intelligence, analyzing the data and then our intensivists giving recommendations back to the hospital*”.

(Int. 4, sec. 4)

“*We are involved in a telemedical development, which is ultimately offered by the German Retirement Insurance (Deutsche Rentenversicherung), via an institute or, no, it is a company that does this, in order to develop a telemedical supervised aftercare in rehabilitation. That is under development. So we already have the initial prerequisites here, but at the moment there is still a bit of a lack of manpower to carry this out*”.

(Int. 12, sec. 5)

Specific implementation steps in the private context included the purchase and use of wearables for vital signs monitoring (Int. 12, sec. 27), as well as the use of a smartwatch with a fall sensor and integrated emergency call function (Int. 1, sec. 6):

“*And here I (shows a ring on his finger), I wear this Ouraring there, you know that too, that also records everything possible. And these are, I think, the first steps*”.

(Int. 12, sec. 27)

“*She wears a watch that can tell if she’s had a fall. And if she lies down and doesn’t move for a while, it detects that, too, and she can use it to make an emergency call*”.

(Int. 1, sec. 6)

#### 3.2.8. Decision

The decision category (*n* = 10) describes whether and how “an individual engages in activities that lead to a choice to adopt or reject the innovation” [19] of telemedicine. This category is illustrated by the following statement:

“*We do home care in the area of telemedicine, so that we can then evaluate and analyze the data from the sensor and the TAN via a dashboard and the cloud*”.

(Int. 6, sec. 4)

None of the respondents rejected telemedical innovations during the interviews. One respondent showed a willingness to offer telemedicine if it was financially worthwhile (Int. 3, sec. 39). Another person reported that their own physician’s office is in the process of implementing video consultation (Int. 1, sec. 27). Another respondent reported a decision to launch another telemedicine product (Int. 2, sec. 29).

#### 3.2.9. Confirmation

The confirmation category (*n* = 8) describes whether an individual, after his or her own experience with telemedicine innovation, actually continues to use the respective innovation or reverses the use because expectations were disappointed. The category includes real as well as hypothetical reasons for adopting telehealth innovation. The reasons given for continuing to use an innovation in telemedicine were simplifications in the professional context (Int. 3, sec. 42), reductions in workload for staff (Int. 3, sec. 44) and innovation profitability (Int. 3, sec. 45). Possible applications for these were electronic appointment scheduling and appointment reminders (Int. 3, sec. 42), and the extension of telemonitoring to all chronically ill patients for postinpatient follow-up care and all fields of application was also mentioned (Int. 13, sec. 27). Another reason given for the continued use of innovations was the vision of wanting to make intersectoral action the standard and thus to underpin it economically (Int. 13, sec. 27).

## 4. Discussion

The results of the expert interviews fit into the current state of research in implementation and innovation science and expand it to include specific aspects of telemedicine innovation in regulated healthcare markets such as Germany. The deductive–inductive analysis, based on Rogers’ adoption theory, was able to extend the existing knowledge about the special significance of adoption experiences in healthcare systems [22,23,24,36] by revealing context-sensitive issues that may act as hidden influencing factors on the diffusion of innovations.

The interviews show that the phenomenon of inconsistent use and attribution of meaning applies not only to the term innovation but also to the concept of telemedicine. Thus, the underlying basic understanding of the definition of telemedicine is the same among all experts and is based on the understanding of the European Commission, according to which “Telemedicine is the provision of healthcare services, through use of ICT, in situations where the health professional and the patient (or two health professionals) are not in the same location” [37]. Despite this common understanding, the responses reveal uncertainties about the technologies and applications involved, as illustrated by the distinction between telematics and telemedicine. One possible reason for this could be the underlying understanding of the term innovation in telemedicine, which was not further queried. A mixture between a telemedicine understanding of “newly perceived” (understanding according to Rogers [19]) and “completely new” (understanding according to Barnett [38]) forms of telemedicine (e.g., telephone consultation vs. video consultation vs. telemonitoring) cannot be ruled out and is indicated in several interviews. Nevertheless, the basic common understanding of the terminology telemedicine allowed an embedding of the analysis in a general context analysis. With this understanding, the outcome categories of persuasion (*n* = 171), knowledge (*n* = 96), implementation (*n* = 19), decision (*n* = 10) and confirmation (*n* = 8) were explored on the basis of E.M. Roger’s adoption theory [19].

Upon further analysis, the most frequently coded segments were the subcodes convictions about the implementation of telemedicine (*n* = 89) (persuasion), aspects of existing telemedicine innovations (*n* = 65) (knowledge), international comparison of Germany’s development (*n* = 50) (persuasion) and aspects of telemedicine in the context of structures in Germany (*n* = 24) (knowledge). The most frequently coded category, persuasion, indicates that respondents’ opinions and attitudes towards the innovations of telemedicine are already well-established. Possible reasons for this could be the pandemic-related information and education campaigns on teleconsultation [39], its billing integration into standard care with the lifting of the previous ban on telemedicine in Germany [40] and a pandemic-related increased sensitivity towards telemedicine/noncontact treatment methods. The statements indicate that physicians and telemedicine experts have a lot of information, opinions and attitudes regarding telemedicine implementation, despite being latecomers to innovation implementation in the healthcare sector.

The attitudes measured in the subcategory convictions regarding the implementation of telemedicine (*n* = 89) cover various topics, such as political will as a prerequisite for the implementation of telemedicine, the existing fears of users, unresolved reimbursement issues or different attributions of the importance of telemedicine, e.g., as process optimization rather than as a new treatment method. Some of these aspects, such as political will, reimbursement issues or fears, are also found in several studies [41,42,43]. Other implementation factors, such as user anxiety, are also discussed in the literature as influencing acceptance and attitudes toward eHealth innovations [44]. This subcategory thus overlaps with previous implementation and acceptance research but differs in its focus on individual attitudes toward telemedicine. Whether and to what extent these attitudes have an effect on the perceived responsibility for implementation could not be clarified. However, there were indications that this is seen at the political level.

In terms of knowledge of existing telemedicine innovations, it became apparent that there was broad knowledge of available technologies and possible applications for telemedicine, although the level of detail differed between respondents. The subcode aspects of telemedicine in the context of structures in Germany (*n* = 24) is closely related to the category persuasion, but it includes the respondents’ knowledge of the specific German structures and framework conditions for the implementation of telemedicine, such as the billing peculiarities of telemedicine-supported functional analyses or the exclusive ban on remote patient treatment that was valid until 2020 [45]. The aspects mentioned here are to be seen in the context of efforts to strengthen “system partnerships between the healthcare industry and the traditional partners (service providers and payers)” [46], which have no tradition in Germany, although they are legally anchored in the Social Code, Book V [46]. They enable cooperation between manufacturers, service providers and payers in the healthcare system and are reflected in pilot projects and integrated care models [47] with the aim of improving the quality of care and treatment, increasing patient satisfaction and reducing treatment costs [48]. If an innovation such as telemedical intervention management for heart failure [49] proves effective, it will be adopted into standard care [50] and will experience the implementation factors described in the literature [41,42,43,51,52,53]. The experts’ statements indicate that the specific healthcare system structures and associated procedures for innovation implementation do not adequately cover the characteristics of telemedicine as a mixture of product, process and service innovation and therefore delay implementation. The extent to which this is a phenomenon specific to the Bismarck system cannot yet be conclusively clarified. However, it appears that the penetration and implementation of innovations such as telemedicine are more difficult in a healthcare system based on self-administration, as is the case in Germany.

The aspects elicited in the subcategory international comparison of Germany’s development (*n* = 50) provide insight into the development of telemedicine in Germany so far in comparison with international healthcare systems from the perspective of the respondents. Distinctive aspects were Germany’s comparatively lagging role in the international field, the national narrative of data protection and the associated perceived “special path” in the implementation of telemedicine in Germany, which have also emerged in the literature as potential barriers to implementation. It also emerged that knowledge about telemedicine is often based on individual, national and international exchanges of knowledge and experience. Overarching information and communication structures for telemedicine appear to be weak in the German healthcare system. Gagnon et al. [54] even reported that organizational and contextual factors can act as support factors for implementation, although these alone cannot predict actual behavior [55]. Against the background of the influence on the perception of and attitude formation toward innovations [22,23,24,36], it is reasonable to assume that the national narrative of data protection and weak information and communication structures have a reciprocal influence on the implementation of telemedicine.

The results of the implementation, decision and confirmation categories with their elicited aspects on implementation steps and the confirmation or rejection of innovation can be understood against the theoretical background of implementation science. They allow theory-based analyses on barriers and facilitators or on the importance of perceptions and individual attitudes towards innovations [18,22,23,24,36]. The different stages of implementation, or even the acceptance or rejection of an innovation, can be understood in this light. For example, resistance to innovative change and insufficient digital literacy [56], as well as fear and anxiety of losing control, can act as behavioral barriers and hinder the implementation of innovations at the individual level [51,57]. During the interviews, telehealth innovations were not rejected by any of the interviewees, and their benefits and potential were recognized. However, the decision to implement had not yet been made by all interviewees. Factors such as financing, acceptance or lack of information were cited as reasons for this, which are already implementation determinants described in the literature [43]. Against the background of the adoption theory according to Rogers [19], the open attitudes towards innovations in telemedicine but only partial implementation indicate that the respondents were in different phases of the individual adoption process. In relation to the respective adoption phase and the underlying individual communication behavior about innovations, there are also indications of the respective adoption type. The lower number of codes for the categories’ implementation, decision and confirmation can thus be an expression of the different adoption processes, as well as the composition of the adoption types.

The resulting topics based on the adoption categories reveal extensive knowledge and diverse attitudes with regard to telemedicine implementation. At the same time, the statements indicate that a mixture of adoption experiences at the individual level and diffusion experiences at the societal level have already taken place, which have shaped the identified opinions and attitudes of the respondents. Nevertheless, it can be assumed that the attitudes identified here at the individual level will also influence the future implementation process, which is why they should be addressed through communication channels as part of the diffusion process.

The aim of this study was to record the individual perceptions and attitudes of medical and telemedicine experts with regard to the implementation of innovations in healthcare systems using the example of telemedicine in Germany. Based on the adoption theory, the aim was to identify topics that act as hidden influencing factors on telemedicine implementation in Germany. Despite careful study planning, the present study has limitations. The small sample of 13 participants does not allow a statistical evaluation of the statements and is therefore not transferable to all physicians and telemedicine experts in Germany. Furthermore, a bias due to socially desirable statements by the interview participants cannot be completely ruled out, despite the assurance of anonymity. In addition, this research work is subject to the qualitative research paradigm of subjectivity due to its methodology [58]. Accordingly, despite compliance with the quality criteria of intracoder reliability and plausibility checks and the rule-based approach according to Kuckarzt [35], the coding may differ from that of other researchers [59], although it can be categorized within the current state of research. Furthermore, it cannot be ruled out that other topics may have been left out, as the data collection was completed after 13 expert interviews in accordance with the principle of theoretical saturation [33,34].

## 5. Conclusions

This study provides insight into how hidden influencing factors for innovation implementation in healthcare systems can be determined on the basis of adoption theory. The results indicate that the implementation of innovations in healthcare systems follows special rules, starting with the difficulty of assigning an innovation in the healthcare sector to a type of innovation and defining it precisely to the mostly hidden attitudes of individuals and complex system structures to the frequently unanswered question of who bears responsibility for implementation in the respective system. Furthermore, the results indicate that digital innovations are perceived as a novelty that has the potential to change the profession of medicine and thus also professional identities in the long term. The extent to which the previous human-centered self-image of healthcare provision is changed by digital innovations and how this affects the further implementation of digital innovations could not be determined and is the subject of further research.

From the explanations presented here, the particular importance of a healthcare-system-wide national knowledge and communication strategy that follows the professional self-image of healthcare provision can be derived. This appears essential in order to develop an implementation narrative in the healthcare system context on the one hand and to create innovation-friendly development structures that take account of the specific character of innovation in the healthcare system on the other.

## Data Availability

The data that support the findings of this study are available on request from the corresponding author. The data are not publicly available due to containing information that could compromise the privacy of research participants.

## Supplementary Materials

The following supporting information can be downloaded at: https://www.mdpi.com/article/10.3390/healthcare12020129/s1, Guideline interview.
