# Supplementary material for: Adoption Processes of Innovations in Health Systems: The Example of Telemedicine in Germany
Source: Healthcare (Basel). 2024 Jan 6;12(2):129. doi: 10.3390/healthcare12020129 (PMC10815117; doi:10.3390/healthcare12020129)
Supplement: Supplementary file 1 [file healthcare-12-00129-s001.zip › healthcare-2719747-supplementary.pdf]

Guideline interview

The framework of the overall interview is the individual expert's understanding of the concept of telemedicine. Based on this, suitable open questions are used to elicit aspects of the (previous and possibly upcoming) adoption process. In the course of the interview, the focus will be on the implementation of new telemedicine technologies or innovations, or a description of the current situation based on the dimensions of the diffusion process according to Rogers.

Due to the pandemic-related situation, the guideline interview is to be conducted remotely via video telephony service provider or by telephone and recorded using audio. The guided interview begins with an open introductory question and is then based on the theoretical frame of reference of Rogers' adoption theory.

Open initial question:

What do you understand by the term telemedicine?

Category-based questions:

Section A: Adoption process

1. In which areas do you deal with telemedicine in your everyday work? What experience have you already gained in this area? (Persuasion/Confirmation)

2. What other types of telemedicine do you know besides those mentioned so far? (Knowledge)

3. In which areas of telemedicine do you see a potential for support in your daily work? (Persuasion)

4. In which areas of telemedicine do you see advantages for your patients? (Persuasion)

5. In your opinion, what conditions need to be created in order to better integrate or implement telemedicine in standard care? (Decision)

Section B: Diffusion process

1. Where, how and to what extent do you find out about the state of telemedicine? (Types of innovations in telemedicine, channels, time/frequency)

2. To what extent do you discuss the development of telemedicine with other experts? Which specialist areas do the dialogue partners come from? (social system)

3. In your opinion, what are the greatest barriers to the expansion of telemedicine in healthcare/hospitalisation/everyday practice?

4. Would you like to include further telemedicine elements in your current healthcare practice?

a. If yes, which ones?

i. What would support you in the implementation or what prerequisites would have to be created?

b. If no, why not?

i. What is currently preventing you from doing so or what conditions would need to be created?

Conclusion:

If you had the opportunity to set up the ideal telemedical care system, what would you do first?
